# Chromosome-level assembly of the Critically Endangered school shark, *Galeorhinus galeus (Linnaeus, 1758)*

**DOI:** 10.1038/s41597-026-07284-2

**Published:** 2026-05-11

**Authors:** Emma de Jong, Floriaan Devloo-Delva, Liam Anstiss, Bruce Deagle, Adrianne Doran, Pierre Feutry, Lauren Huet, Katherine Ollerhead, Lara Parata, Jayson Semmens, Shannon Corrigan

**Affiliations:** 1 Minderoo OceanOmics Centre at UWA, Oceans Institute, University of Western Australia, Crawley, WA Australia; 2 Australian National Fish Collection, NCMI, CSIRO, Hobart, TAS Australia; 3 Environment, CSIRO, Hobart, TAS Australia; 4 Fisheries and Aquaculture Centre, Institute for Marine and Antarctic Studies, University of Tasmania, Hobart, TAS Australia; 5 Minderoo Foundation, Perth, WA Australia

**Keywords:** Genome, Genome informatics

## Abstract

The school shark, *Galeorhinus galeus* (Linnaeus, 1758), is the only extant species within its genus and is widely distributed in benthopelagic habitats of temperate coastal regions. Fishing pressure has caused global population declines by more than 80%, resulting in the species being listed as Critically Endangered. Here, we present the first chromosome-level, haplotype-phased reference genome assemblies for *G. galeus*, including assemblies of both male and female individuals. The 4.81 Gbp male genome was assembled from PacBio HiFi and Dovetail Omni-C proximity ligation sequencing data, and the 4.52 Gbp female genome was assembled from PacBio HiFi data scaffolded via homology to the curated male genome. Both genomes are >92% BUSCO complete and contain ~60–63% repeat content. Analysis of methylation levels and single nucleotide polymorphism density across chromosomes revealed several regions where high SNP density co-located with relative hypomethylation, indicating a reciprocal relationship between these two features. These genomes are a valuable resource enabling future genomic studies to inform population management and conservation strategies for this Critically Endangered species.

## Background and Summary

**Fig. 1:**
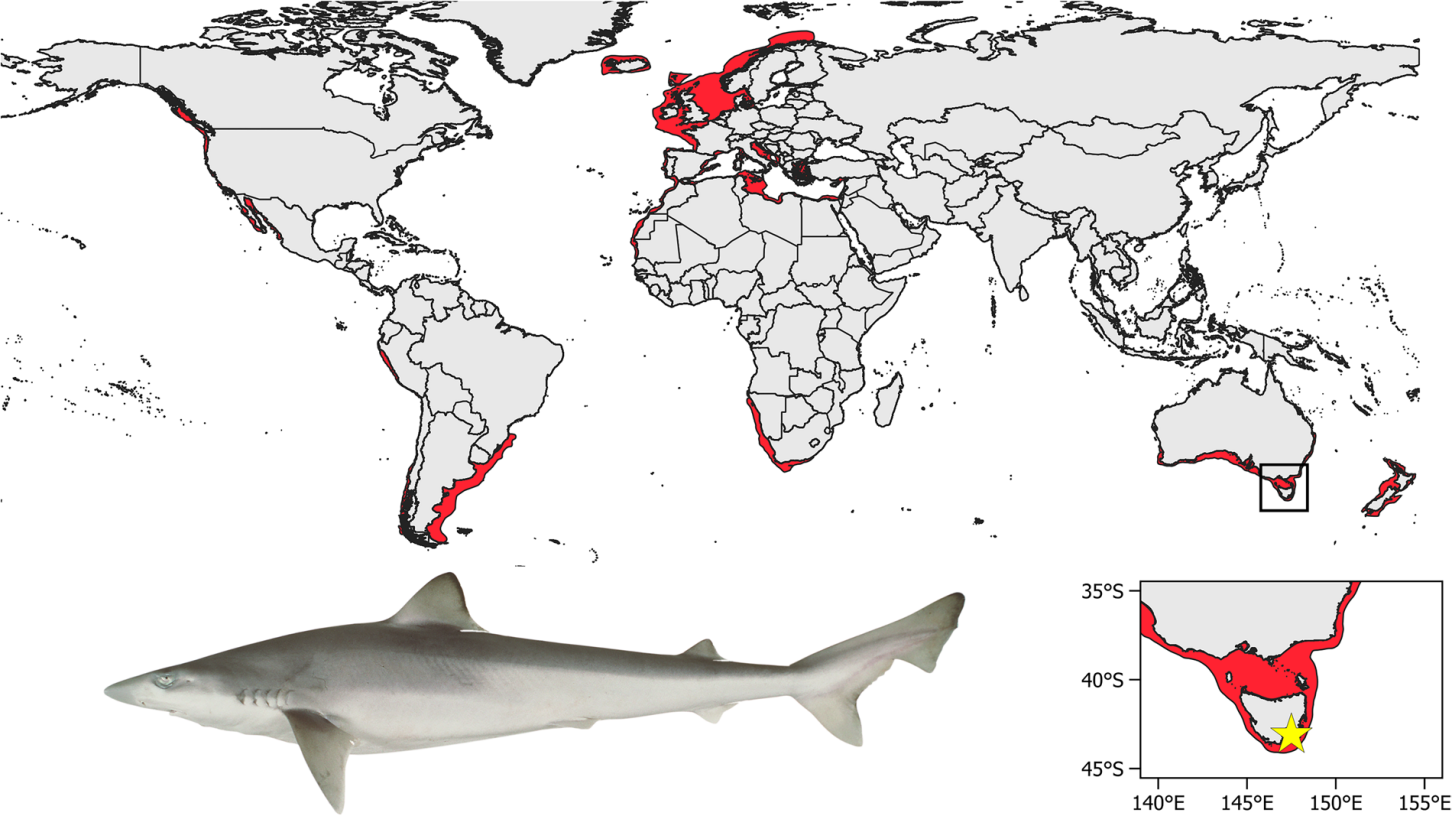
(**A**) Global distribution of the *Galeorhinus galeus* and sampling location of the specimens (indicated with yellow star). (**B**) Image of the school shark from the CSIRO Australian National Fish Collection (Registration number: CSIRO H 4153-01).

The school shark (a.k.a. tope or soupfin shark, *Galeorhinus galeus*; Linnaeus, 1758) can reach up to 200 cm total length and is found in benthopelagic habitats across the temperate waters of most oceans (Fig. 1A). The school shark is the only extant species of genus Galeorhinus. While the school shark can travel thousands of kilometres^1^, large oceanic distances are considered barriers to genetic connectivity and six populations are recognised globally: Northeast Atlantic (including the Mediterranean Sea), southern Africa, Southwest Atlantic, Northeast Pacific, Southeast Pacific, and Australasia^2^. A global assessment of conservation status by the IUCN Red List of Threatened Species categorised the school shark as Critically Endangered^2^. Its importance for fisheries worldwide, both as target species and as bycatch, combined with its slow growth and late maturity, has led to significant population declines (>80% in the last 79 years)^2,3^. Within Australia, only few nursery areas sustain sufficient recruitment to rebuild the population^4^. One of these nursery areas, the Pittwater Estuary in southern Tasmania, has maintained a steady recruitment since the 1990s but recently it has seen increasing impacts of marine heatwaves as the East Australia Current extends further south^5,6^. Given the species’ evolutionary distinctiveness, economic/ecological importance and high risk to extinction, we constructed a chromosome-level reference genome to understand its evolutionary history and support genome-enabled research that can inform ongoing conservation management strategies. Assemblies from both male and female individuals were >92% BUSCO complete, with scaffold NG50 values > 55 Mbp, and QV scores > 56.

## Methods

### Sample collection

Two juvenile school sharks (one male and one female) were collected from the Pittwater Estuary, Tasmania, Australia (Fig. 1B). Both individuals were caught using bottom-set long lines under Department of Primary Industries Parks Water and Environment permits (#11055 & #21080) and with approval from the University of Tasmania Animal Ethics Committee (#A0011882).

### Genomic DNA extraction, library construction, and sequencing

High molecular weight (HMW) genomic DNA was extracted in triplicate from approximately 20 mg of heart tissue each from the male individual using the Nanobind tissue kit (PacBio, CA, USA). Tissue was homogenised using the TissueRuptor II (QIAGEN, Hilden, Germany), with cell lysis and DNA isolation performed as per PacBio “Extracting HMW DNA from skeletal muscle using Nanobind” procedure (102-579-200, Dec 2022). DNA concentration was quantified using a Qubit 3 Fluorometer with the Qubit dsDNA Broad-Range Assay Kit (Thermo Fisher Scientific, MA, USA), and a NanoDrop One (Thermo Fisher Scientific, MA, USA). The fragmentation profile of the HMW DNA was assessed using a Femto Pulse with the Genomic DNA 165 kb kit (Agilent, CA, USA). Three PacBio HiFi SMRTbell® libraries were prepared using the PacBio SMRTbell® prep kit 3.0 according to manufacturer’s instructions. These libraries had mean fragment lengths of 14,664 bp, 18,261 bp and 21,086 bp, and were sequenced over five SMRT cells on a PacBio Revio sequencing instrument, collectively producing 344.57 Gbp of HiFi data with an average read length of 12.94 kbp (Table 1). Genomic DNA from the female individual was extracted from muscle tissue, using the Nanobind tissue kit. PacBio HiFi library preparation was performed at the Australian Genome Research Facility (AGRF Ltd., Brisbane, Australia) and sequenced on six SMRT cells on the PacBio Sequel II.

**Table 1:** Raw sequencing data metrics for *Galeorhinus galeus* specimens.

Technology	Male (sGalGal3)		Female (sGalGal4)	
	Data (Gbp)	Coverage (X)	Data (Gbp)	Coverage (X)
**PacBio HiFi**	344.57	84.45	170	34.00
**Hi-C**	288.12	70.61	N/A	N/A
**RNA-Seq**	93.72	55.46	N/A	N/A

### Chromatin conformation capture proximity ligation library construction and sequencing

Liver tissue from the male individual was homogenised using a Biomasher II on dry ice with the PowerMasher II (Nippi Inc., Tokyo, Japan) and processed using the Dovetail® Omni-C® Kit according to the manufacturer’s protocol (Cantata Bio, CA, USA). Briefly, the chromatin was fixed in the nucleus with disuccinimidyl glutarate (DSG) and formaldehyde. Cross-linked chromatin was then digested *in situ* with DNase I. Cells were then lysed with SDS and extracted chromatin fragments were bound to Chromatin Capture Beads. Chromatin ends were then repaired and ligated to a biotinylated bridge adapter followed by proximity ligation of adapter-containing ends. After proximity ligation, the crosslinks were reversed, the associated proteins were degraded, and the DNA was purified then converted into a sequencing library using Illumina-compatible adaptors. Biotin-containing fragments were isolated using streptavidin beads prior to PCR amplification. In total, four libraries were generated from two liver aliquots. Each library was shallow sequenced on an Illumina iSeq 100 platform using a 2 × 150 bp paired-end run, to confirm sufficient library complexity prior to deep sequencing. Deep sequencing of the four libraries was then carried out on an Illumina Novaseq. 6000 platform with a 2 × 150 bp pair-end run configuration to generate 288.12 Gbp of high-throughput chromosome conformation data (Table 1)^7^.

### RNA extraction, library construction and sequencing

Total RNA was extracted from the muscle, liver, heart and gonad tissues of the male individual using the Monarch Total RNA Miniprep Kit (New England Biolabs, USA). RNA was pooled at equimolar amounts and the Iso-seq library was prepared with the PacBio Kinnex full-length RNA kit at AGRF (Brisbane, Australia). The library was sequenced on one SMRT cell on the PacBio Revio, yielding 93.72 Gbp of HiFi data with an N50 length of 11.35 Kbp after quality filtering with the ccs v8.0.0 and SMRT Link v13.0 tools.

### Genome assembly and quality assessment

**Fig. 2:**
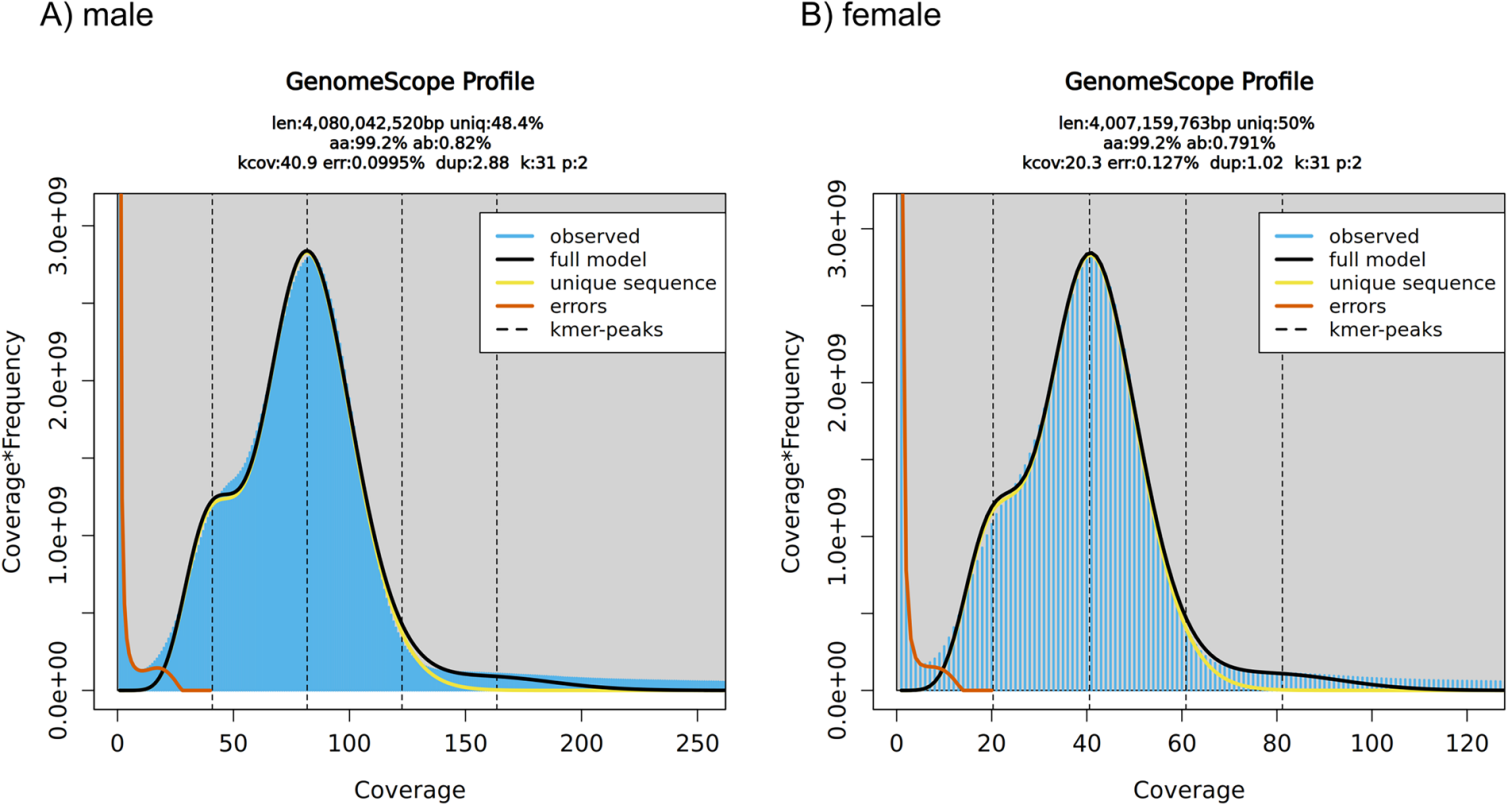
GenomeScope2.0 transformed linear plots for *Galeorhinus galeus*. Analysis of (**A**) male, and (**B**) female specimen data generated from PacBio HiFi reads.

Raw HiFi reads were screened for adapter contamination using HiFiAdapterFilt v.2.0^8^, and passed through Meryl v1.3^9^ (k = 31) and GenomeScope2 v2.0^10^ to generate genome profiles and size estimates for both specimens (Fig. 2). A haplotype-phased chromosome-level reference genome for *G. galeus* was assembled from HiFi and Hi-C data derived from the male specimen (sGalGal3) following Vertebrate Genomes Project workflows^11,12^. Briefly, contig-level assemblies were generated with Hifiasm v0.19.8^13,14^ in Hi-C mode, and then scaffolded to chromosome-level with YAHS v1.2a.2^15^ based on Hi-C read alignments, which were produced following the Omni-C mapping pipeline^16^. The scaffolded haplotype 1 and 2 assemblies were then concatenated for dual manual curation using PretextMap v0.1.9 (https://github.com/sanger-tol/PretextMap) and PretextView v0.2.5 (https://github.com/sanger-tol/PretextView). In the absence of Hi-C data for the female specimen (sGalGal4), the curated haplotype 1 male assembly (sGalGal3.hap1) was used as input for homology-based scaffolding of the female contig-level assemblies with RagTag v2.1.0^17^. The resulting chromosome-level female assemblies were then subjected to the same manual curation process using PretextMap and PretextView. All assemblies underwent decontamination using FCS-GX v0.5.4^18^ and Tiara v1.0.3^19^ to eliminate sequences originating from foreign organisms or mitochondria. The final curated genome assemblies were quality-assessed using gfastats v1.3.6^20^, analysis of Benchmarking Universal Single-Copy Orthologs (BUSCO) v5.4.7^21^ (vertebrata odb10) and Merqury v1.3^9^.

### Repeat annotation

**Fig. 3:**
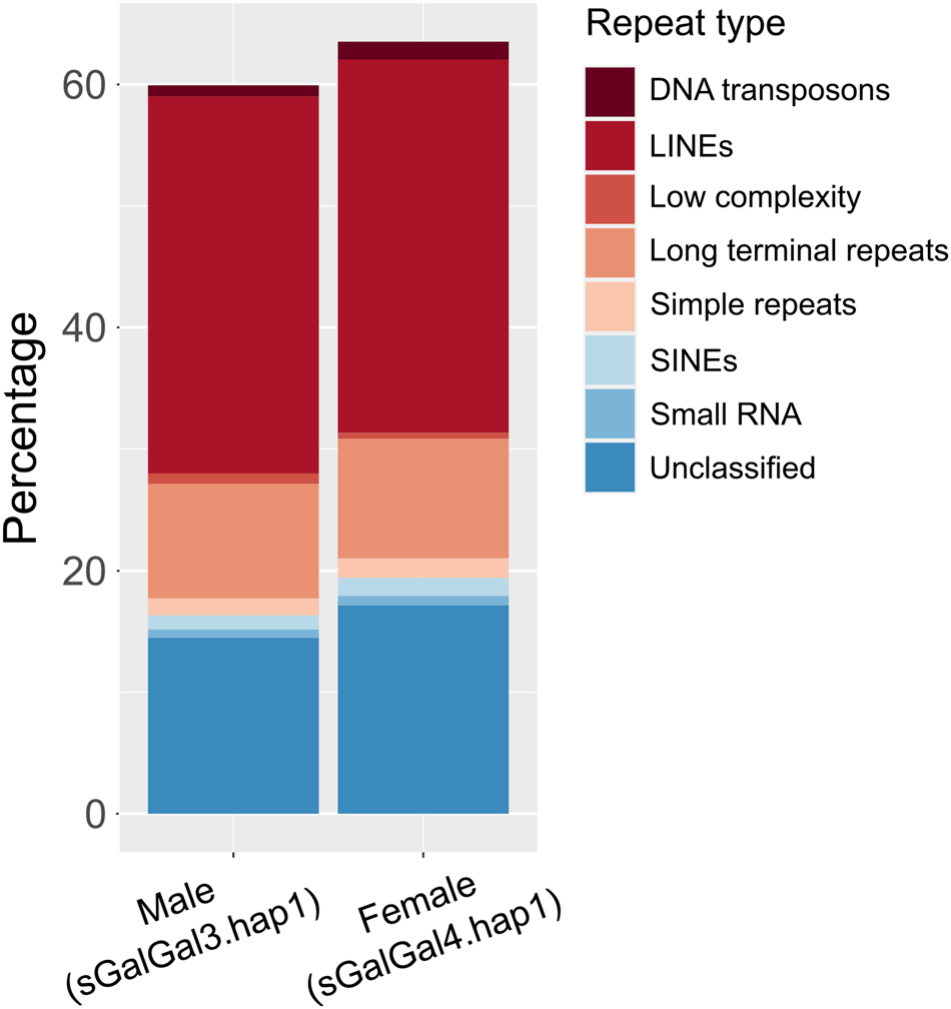
The percentage of major repeat types in male and female haplotype 1 assemblies. LINEs, long interspersed nuclear elements; SINEs, short interspersed nuclear elements.

A custom repeat library was generated with RepeatModeler (v2.0.5)^22^ using the curated haplotype 1 assemblies for each of the male and female *G. galeus* specimens. The resulting libraries were used to generate repeat statistics using RepeatMasker (v4.1.5)^22^. Overall, RepeatMasker identified 2,897.95 and 2,858.72 Mbp of repetitive elements in the male and female assemblies, covering 60.17% and 63.17% of the genomes, respectively (Fig. 3, Table 2), in line with proportions of repeat content observed in other chondrichthyans: 50.34% of the *Rhincodon typus* (whale shark) genome^23^, 58.5% of the *Carcharodon carcharias* (white shark) genome^24^.

**Table 2:** Repetitive elements of the final male and female haplotype 1 assemblies.

	Number of elements		Length (Mbp)		% in genome	
	Male	Female	Male	Female	Male	Female
**Short interspersed nuclear elements (SINEs)**	479,025	549,432	56.56	67.51	1.17	1.49
**Long interspersed nuclear elements (LINEs)**	3,181,515	3,220,598	1495.64	1390.87	31.05	30.73
**Long terminal repeats (LTRs)**	1,267,233	1,337,772	452.43	443.86	9.39	9.81
**DNA transposon**	172,498	145,849	44.32	66.3	0.92	1.47
**Rolling circles**	11,060	1,665	0.93	0.26	0.02	0.01
**Unclassified**	3,951,323	3,672,104	697.23	775.46	14.48	17.14
**Small RNA**	234,297	329,645	32.87	35.81	0.68	0.79
**Satellites**	35,206	7,814	4.12	2.13	0.09	0.05
**Simple repeats**	312,194	309,447	66.85	72.49	1.39	1.6
**Low complexity**	57,576	53,990	40.75	21.96	0.85	0.49

### Gene annotation

The PacBio Kinnex Iso-seq yielded 8,167,568 HiFi reads (64,781,490 segmented reads) which were refined through the lima v2.12.0 and IsoSeq v4.2.0 tools for the removal of cDNA primer and chimeric or polyA-tailed sequences. The resulting 63,780,987 Full-Length Non-Chimeric reads (average length of 1,310 bp), together with RNAseq data from *Mustelus asterias* (ERR13148280), *M. griseus* (DRR400782 and DRR400783), and *M. canis* (SRR3632060), were used as input to predict protein-coding genes in the masked genome with the EGAPx pipeline v0.4.1-alpha (https://github.com/ncbi/egapx). A total of 25,471 genes were annotated, 22,459 of which were protein-coding, with BUSCO results showing that 95.0% were complete BUSCOs (80.9% complete and single-copy, 14.1% complete and duplicated).

### Pangenome, structural variation and methylation levels

**Fig. 4:**
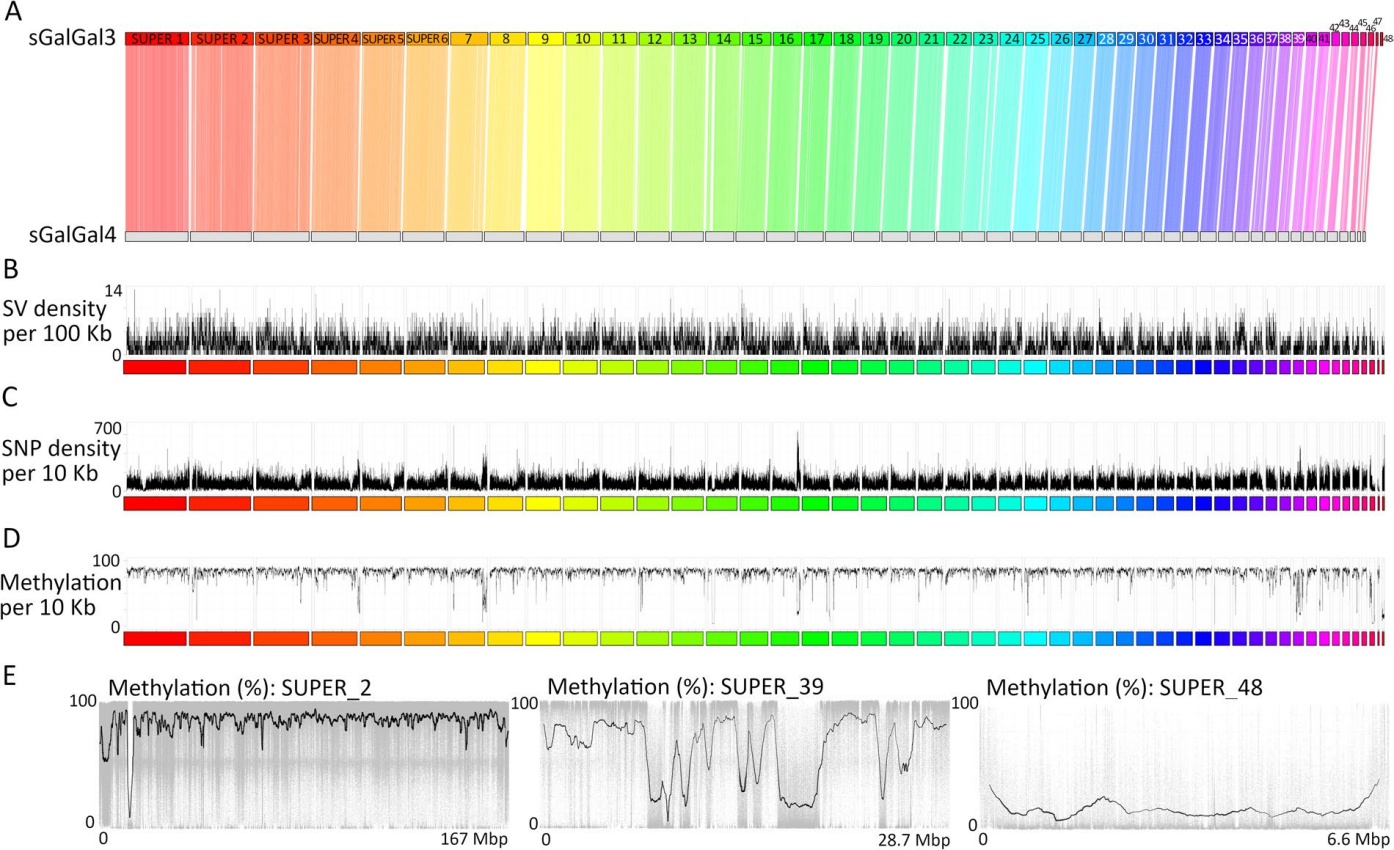
Genomic variation of *Galeorhinus galeus*. (**A**) Cactus pangenome synteny plot of the male (sGalGal3) and female (sGalGal4) haplotype 1 assemblies. Synteny thresholds are based on the phylogenetic distance, resulting in a mash distance of 0.0046 for sGalGal4. (**B**) Structural variant (SV) density in sGalGal3 and sGalGal4 per chromosome over a 100 Kbp sliding window. (**C**) SNP density in sGalGal3 and sGalGal4 per chromosome over a 10 Kbp sliding window. (**D**) CpG methylation percentage in sGalGal3 per chromosome over a 10 Kbp sliding window. (**E**) CpG methylation percentage of chr2, chr39 and chr48. Each grey dot is the methylation per CpG site, and the solid line represents the average over a 10 Kbp sliding window.

**Fig. 5:**
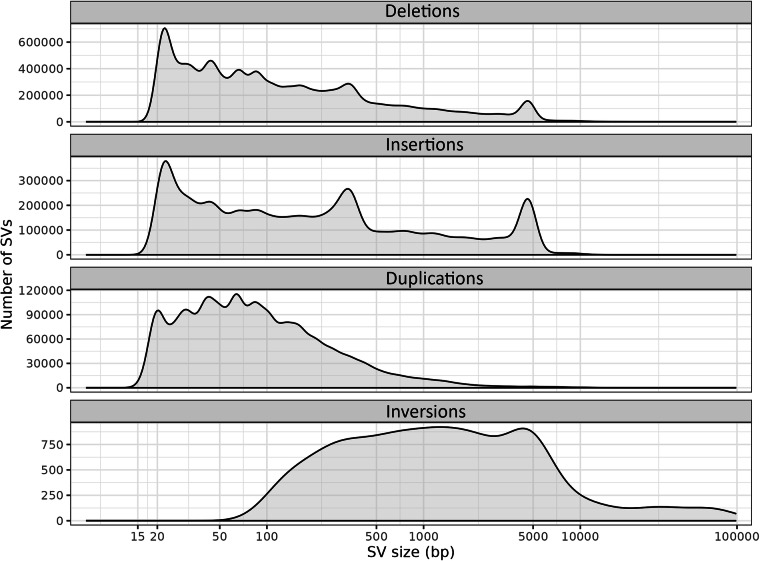
Size distribution of structural variants (SVs) of *Galeorhinus galeus* (sGalGal3 and sGalGal4). The structural variants include deletions, insertions, duplications, and inversions. The x-axis represents the size of these variants on a logarithmic scale.

Haplotype 1 of both the male and female individuals were combined with Minigraph-Cactus pangenome v2.9.8 for whole genome synteny analysis (Fig. 4A). The PacBio HiFi data from both individuals were aligned to the sGalGal3.hap1 assembly to assess single nucleotide polymorphisms (SNPs; Deepvariant v1.4.0), structural variants (SVs; pbsv v2.11.0), and methylation levels (pb-CpG-tools v3.0.0; Fig. 4B-E). This resulted in 22,170,291 biallelic SNPs and 1,101,052 SVs (585,636 deletions, 389,571 insertions, 124,169 duplications, 1,676 inversion and no translocations; Fig. 5). On average, the genome exhibited 71.8 SNPs per 10 Kbp and 2.0 SVs per 100 Kbp, but regions at centromeres had lower SNP and SV densities (<5 SNPs per 10 Kbp, Fig. 4B-C). We identified 101,478,655 CpG sites which had an average methylation level of 72% (Fig. 4D). Most chromosomes had high methylation levels, but interestingly, large sections of chr2, chr7, chr14, chr16, chr39, chr46 and chr48 exhibited very low methylation levels (<60%; Fig. 4C-D). Remarkably, these hypo-methylated regions had high SNP densities (>120 SNPs per 10 Kbp, Fig. 4B-C).

## Data Records

All sequencing data for sGalGal3 are deposited in the European Nucleotide Archive under study accession ERP185579^25^. Haplotype 1 and 2 assemblies are available under accessions GCA_977071755^26^ and GCA_977071745^27^, respectively, and transcriptome data is available under accession SRP564200^28^. Sequencing data, haplotype 1 and haplotype 2 assemblies for sGalGal4 are available in the European Nucleotide Archive under accessions SRP564421^29^, GCA_977109155^30^ and GCA_977109145^31^, respectively. All sequencing data is provided in standard gzip compressed fastq format: details relating to library preparation and sequencing technology are described within the ENA Experiment accession links for each file. Assemblies are provided as standard fasta format files where all chromosome sequences are labelled with *chr* suffixes, unlocalised chromosomal sequences are labelled with *unloc* suffixes, and all unplaced scaffold sequences are labelled with the *Scaffold* prefix. The annotation results including functional predictions, coding sequences, protein sequences and a BUSCO report, along with methylation and variant data are available under the CSIRO Data Access Portal^32^ and can be accessed here: https://data.csiro.au/collection/csiro:73881. Alternatively, the data is accessible from the OceanOmics S3 data repository using the AWS CLI (no AWS account required) with the following commands:


aws s3 ls–no-sign-request s3://minderoo-oceanomics/species/Galeorhinus_galeus/sGalGal3/annotation/–recursiveaws s3 ls–no-sign-request s3://minderoo-oceanomics/species/Galeorhinus_galeus/sGalGal3/methylation/–recursiveaws s3 ls–no-sign-request s3://minderoo-oceanomics/species/Galeorhinus_galeus/variants/–recursive


## Technical Validation

### Molecular validation of nominal specimen identity

The collected specimens were visually identified by experts. Samples collected from Tasmania are expected to share the same genetic background as animals from southern Australia and New Zealand^33^. No cryptic diversity has been identified to date. The nominal species identification was verified by assembling whole mitochondrial genomes from both male and female PacBio HiFi data, using MitoHiFi (v3.0.0). Reads were mapped to the available mitogenomes of a South African (ON652874) and Portuguese (OR702582) individual. This yielded an 18,736 bp mitogenome (38,859 mapped reads and 11,611.1 X coverage) for the male and a 18,735 bp mitogenome (6,151 mapped reads and 2,140.42 X coverage) for the female. The minimum pairwise identity between these four individuals was 99.4%, thereby confirming the species identity and lack of cryptic mitogenome diversity.

### Quality control of nucleic acids and extracts

The quality and quantity of extracted DNA for the male individual was assessed using a Qubit 3 Fluorometer, a NanoDrop One Spectrophotometer and an Agilent Femto Pulse. The triplicate DNA extracts were determined to be of high molecular weight, with concentrations of 66.3 ng/µl, 22.5 ng/µl and 159 ng/µl, a Qubit:NanoDrop ratios of more than 0.89 and average lengths of 42,389, 21,681 bp and 110,098 bp. Quality of the Omni-C lysates were assessed using a Qubit 3 Fluorometer and 4150 TapeStation system. The Omni-C lysates had yields of 11,075 ng and 5,250 ng with 90.25% and 92.74% Chromatin Digestion Efficiency (CDE) metrics between the recommended range of 100–2500 bp. RNA quality and quantity was determined using a Qubit 3 Fluorometer and a 2100 Bioanalyzer (Agilent Technologies). The pooled RNA extractions had a concentration of 50 ng/µl and an RNA integrity number (RIN) of 5.5.

### Evaluation of the genome assembly

**Fig. 6:**
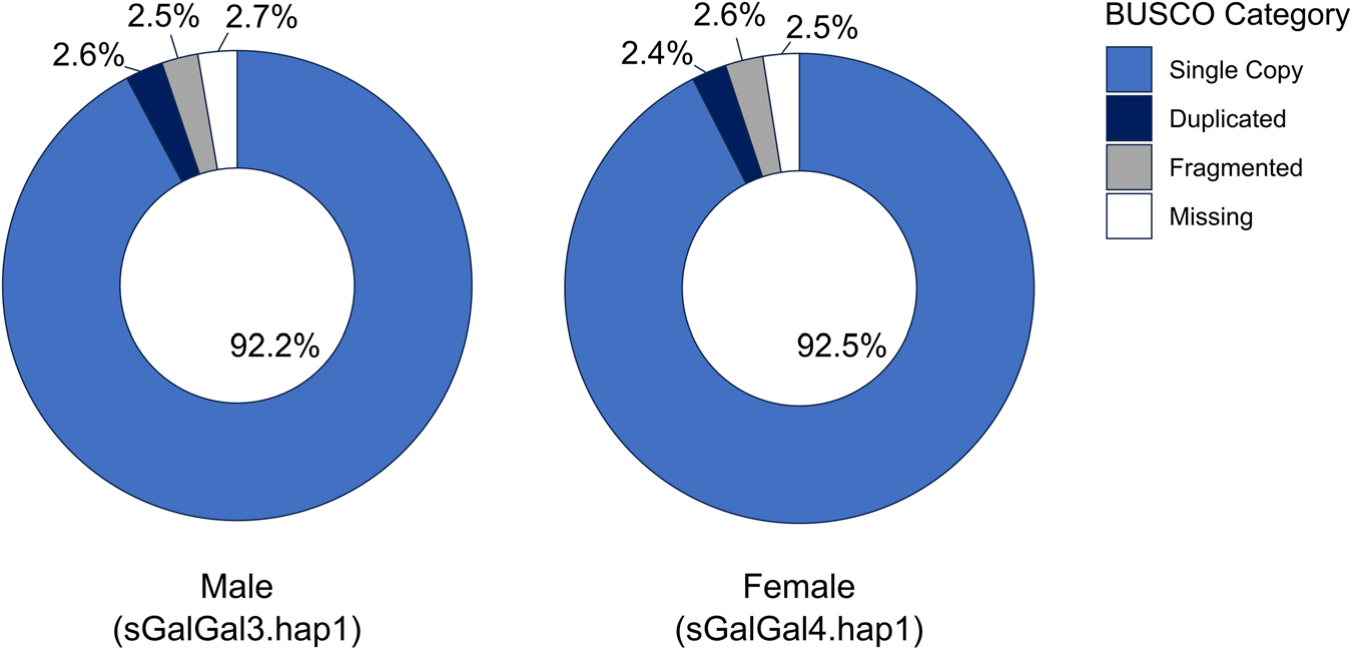
Gene completeness of haplotype 1 assemblies. Donut plots indicating the proportions of single-copy, duplicated, fragmented, and missing Benchmarking Universal Single-Copy Orthologs (BUSCO) genes.

The male and female haplotype 1 assemblies were 4,816,164,632 and 4,525,537,202 bp long, respectively. The genome size was similar to estimates of the closely-related *Mustelus asterias* (GCA_964213995, 3.68 Gbp), but smaller than DNA content estimates based on Feulgen Densitometry (C-value = 8.65 pg)^34^. No karyotype data was available for this species. Quality assessment of the genomes with gfastats v1.3.6 indicated that the contiguity of each genome was at least 55.15 Mbp (NG50), with at least 64.2% of sequences assigned to chromosomes (Table 3). In terms of completeness, both the male and female haplotype assemblies contained approximately 92% of the 3,354 vertebrata BUSCO genes (Fig. 6) and were over 98% complete based on k-mer analysis using Merqury v1.3. These metrics were similar to recently published reference genomes for the epaulette shark (*Hemiscyllium ocellatum*)^35^, the whitespotted bamboo shark (*Chiloscyllium plagiosum*)^36^, and the spiny dogfish (*Squalus acanthias*)^37^. Consensus accuracy, as measured by QV scores using Merqury v1.3, exceeded 60 for the male assembly and 56 for the female assembly (Table 3).

**Table 3:** Assembly metrics for the male and female *Galeorhinus galeus* reference genomes.

	VGP* standards (7.c.P6.Q50.C95)	Male (sGalGal3)		Female (sGalGal4)	
		Haplotype 1	Haplotype 2	Haplotype 1	Haplotype 2
**Total length (Mb)**	—	4816.16	4102.52	4525.54	5724.09
**Number of contigs**	—	10,777	12,335	18,148	23,875
**Number of scaffolds**	—	7,118	10,251	13,286	18,529
**Contig NG50 (Mb)**	>10	1.74	1.66	0.9	0.75
**Scaffold NG50 (Mb)**	=Chr. NG50	55.14	9.1	61.03	31.62
^†^**BUSCO complete (%)**	>95	92.2	91.2	92.5	92.6
**Gaps per Gb**	<200 per Gb	892	508	1213	1334
***Base-pair proportion of the assembly assigned to chromosomes (%)****.*	>95	64.2	50	69.5	55.6
^‡^**QV**	>50	60.05	60.4	56.5	56.5
		60.2		56.5	
^‡^**Merqury completeness (%)**	>95	86.6	88.1	86.9	88.9
		98.5		98.6	

*VGP; Vertebrate Genomes Project^12^. †Values represent single-copy complete BUSCO genes. ‡Merged columns reflect combined (i.e., haplotype 1 and haplotype 2) assembly statistics.

### Ethics declarations

These samples were collected under Department of Primary Industries Parks Water and Environment permit (#11055 & #21080) and with approval from the University of Tasmania Animal Ethics Committee (#A0011882).

## Data Availability

All sequencing data for sGalGal3 are deposited in the European Nucleotide Archive under study accession ERP185579^25^. Haplotype 1 and 2 assemblies are available under accessions GCA_977071755^26^ and GCA_977071745^27^, respectively, and transcriptome data is available under accession SRP564200^28^. Sequencing data, haplotype 1 and haplotype 2 assemblies for sGalGal4 are available in the European Nucleotide Archive under accessions SRP564421^29^, GCA_977109155^30^ and GCA_977109145^31^, respectively. Additionally, genome assemblies and associated annotation, methylation, variant, and quality control data are publicly available at the following AWS Registry of Open Data page: https://registry.opendata.aws/oceanomics/, Data can be accessed via the CSIRO Data Access Portal here: https://data.csiro.au/collection/csiro:73881^32^ and the AWS CLI (no AWS account required) with the following commands. To list all files: aws s3 ls–no-sign-request s3://minderoo-oceanomics/species/Galeorhinus_galeus/–recursive, To download all files: aws s3 cp–no-sign-request s3://minderoo-oceanomics/species/Galeorhinus_galeus/.–recursive

## Code availability

No custom scripts or code were used in the validation of the dataset. The data analyses used standard bioinformatic tools specified in the methods. All code associated with the reference genome assembly workflow is available on available on GitHub: https://github.com/Minderoo-OceanOmics-Centre-UWA/OceanOmics-OceanGenomes-ref-genomes, https://github.com/FloriaanDD/galeorhinus_galeus_genome.
